# Mass Spectrometry Based Metabolomics Comparison of Liver Grafts from Donors after Circulatory Death (DCD) and Donors after Brain Death (DBD) Used in Human Orthotopic Liver Transplantation

**DOI:** 10.1371/journal.pone.0165884

**Published:** 2016-11-11

**Authors:** Olga Hrydziuszko, M. Thamara P. R. Perera, Richard Laing, Jennifer Kirwan, Michael A. Silva, Douglas A. Richards, Nick Murphy, Darius F. Mirza, Mark R. Viant

**Affiliations:** 1 School of Biosciences, University of Birmingham, Edgbaston, Birmingham, B15 2TT, United Kingdom; 2 The Liver Unit, Queen Elizabeth Hospital Birmingham, Edgbaston, Birmingham, B15 2TH, United Kingdom; 3 The Department of Pharmacology, School of Medical and Dental Sciences, University of Birmingham, Edgbaston, Birmingham, B15 2TT, United Kingdom; 4 Department of Critical Care and Anaesthesia, Queen Elizabeth Hospital Birmingham, Edgbaston, Birmingham, B15 2TH, United Kingdom; University of Pittsburgh, UNITED STATES

## Abstract

Use of marginal liver grafts, especially those from donors after circulatory death (DCD), has been considered as a solution to organ shortage. Inferior outcomes have been attributed to donor warm ischaemic damage in these DCD organs. Here we sought to profile the metabolic mechanisms underpinning donor warm ischaemia. Non-targeted Fourier transform ion cyclotron resonance (FT-ICR) mass spectrometry metabolomics was applied to biopsies of liver grafts from donors after brain death (DBD; n = 27) and DCD (n = 10), both during static cold storage (T_1_) as well as post-reperfusion (T_2_). Furthermore 6 biopsies from DBD donors prior to the organ donation (T_0_) were also profiled. Considering DBD and DCD together, significant metabolic differences were discovered between T_1_ and T_2_ (688 peaks) that were primarily related to amino acid metabolism, meanwhile T_0_ biopsies grouped together with T_2_, denoting the distinctively different metabolic activity of the perfused state. Major metabolic differences were discovered between DCD and DBD during cold-phase (T_1_) primarily related to glucose, tryptophan and kynurenine metabolism, and in the post-reperfusion phase (T_2_) related to amino acid and glutathione metabolism. We propose tryptophan/kynurenine and S-adenosylmethionine as possible biomarkers for the previously established higher graft failure of DCD livers, and conclude that the associated pathways should be targeted in more exhaustive and quantitative investigations.

## Introduction

Small (<1,000 Da) metabolites regulate cell signalling, cell-to-cell communication and energy transfer amongst other cellular processes, and are the first biochemicals to respond to internal or external stimuli. This responsiveness makes the metabolome an informative measure of the cell’s dynamic state, a property that has led to a considerable and growing interest in the application of metabolomics in the health sciences. In parallel, metabolomics has begun to be used in clinical solid organ transplantation [1,2] where it holds considerable promise for the discovery of biomarkers to predict poor graft function or patient survival, as well as to elucidate the molecular mechanisms underlying pathophysiological processes such as during graft dysfunction, injury or rejection [3]. Beginning with early studies using ^1^H-NMR spectroscopy, the applications of metabolomics in transplantation have expanded in more recent publications to using mass spectrometry based profiling of poor allograft function [4]. Case studies using NMR spectroscopy identified six potential metabolic biomarkers that were distinctive of a non-functional liver[5,6]. Meanwhile a metabolomics assessment in cirrhotic patients by ^1^H-NMR spectroscopy linked elevated levels of high-density lipoproteins and phosphocholine with mild chronic liver failure whereas elevated levels of lactate, pyruvate, glucose and creatinine were associated with severe chronic liver failure (CLF). This study provided new insights to hepatic functional impairment in cirrhosis as well as showed an alternative approach to evaluate the severity of CLF, a key aspect in therapeutic decision making [7]. Furthermore, analyses of human bile during liver transplantation by capillary electrophoresis showed distinct metabolic fingerprints in donors and recipients [8]. Whereas more recent publication has been focussed on much larger group of liver grafts belonging to two distinct organ donor sources, namely the cadaveric donation after circulatory death (DCD), and donation after brain death (DBD) and applied lipidomics studies identify markers of early allograft dysfunction [9].

While liver transplantation is well established as the treatment of choice for many indications, the ever growing number of patients listed for transplantation has outweighed the supply of cadaveric organs, leading to greater disparity between supply and demand. As a result, most transplant programmes turned to using marginal organs, in particular liver grafts obtained by donation after circulatory death (DCD), to supplement more traditional donation after brain death (DBD) [10]. Despite rigorous donor selection criteria and best efforts to match a DCD graft with an optimal recipient, the results of the DCD transplantations remain inferior to the standard DBD transplantations and include increased occurrence of primary non-function (PNF) and biliary complications in the immediate post-operative period as well as inferior long-term graft survival rates [11–13]. These complications have been initially associated with donor warm ischaemia (Greek; *isch–*restriction, *hema—*blood), typical of DCD grafts during the surgical withdrawal phase. This phase is of variable and unpredictable duration which each DCD donor undergoes once the life support therapy is halted to allow natural passage to circulatory death [14]. Furthermore, grafts obtained from deceased donors are typically stored on ice (at a temperature between 0–4°C) before these organs are transplanted to the recipient, and this phase of cold storage further aggravates graft injury due to ischaemia [15]. Once the blood supply is reconnected within the recipient (both DCD and DBD) to the transplanted organs (termed reperfusion), further organ injury occurs through a process known as ischaemia-reperfusion or preservation-reperfusion injury. In this multifactorial process the reactive oxygen species generated in the organ during warm ischaemia initiate a cellular cascade leading to inflammation, and in severe cases to organ failure termed as PNF [16,17].

Identifying the metabolic differences between DCD and DBD liver grafts could significantly improve current clinical practise by defining biomarkers that are predictive of poor graft function prior to transplantation. Selection of grafts from donors that exhibit such metabolic biomarkers could assist in clinical decision making and the exclusion of those organs from transplantation, thereby preventing the adverse clinical sequelae after transplantation. This would also help those transplant programs that are reliant on cadaveric donor organs for transplantation by preventing the necessity to perform re-transplant operations on those who had failed liver grafts, minimising the burden and demand for organs. Furthermore, identifying the metabolic differences between DCD and DBD liver grafts could help to identify the metabolic modifications of livers prior to and after the organ procurement from the donor that would improve the organ quality, an approach called metabolic therapy. This approach is justified by the nature of current organ donation practice. In the United Kingdom alone there has been a steep rise in DCD donations in recent years, however only 27% of liver grafts from these donors are used in clinical transplantation [18] with approximately 8% of these being excluded due to the high risk of PNF. In addition, the majority of grafts are not even procured due to increased time elapsed between the treatment withdrawal phase and circulatory death, which is beyond the currently accepted criteria of donor warm ischaemia time (30 minutes). Relating the metabolic profiles of DCD and DBD liver grafts to the outcome of the transplantation could supplement and expand the traditional methods to predict organ function, in particular early on during the transplantation procedure.

Previously we reported a pilot study that demonstrated the potential of Fourier transform ion cyclotron resonance (FT-ICR) mass spectrometry to detect a few thousand metabolic features (or peaks) in biopsies obtained from liver grafts in the cold and post-reperfusion phases of orthotopic liver transplantation (OLT)[19]. We observed and characterized changes in multiple metabolic pathways showing a rapid resumption of biochemical function within the grafts following reperfusion [19]. Here, we expand considerably upon this initial investigation, specifically with the aim to investigate and characterize the metabolic differences between DCD and DBD liver grafts at two key phases of the liver transplantation, the cold storage phase (T_1_) and post-reperfusion phase (T_2_). We seek to reveal the underlying metabolic pathways associated with the clinical observation of reduced success of the DCD grafts.

## Materials and Methods

This study was approved by the South Birmingham Regional and National Research Ethics committee. The study was aimed at discovery of biomarker differences in the liver grafts used in the clinical orthotopic liver (OLT) setting with possibility of identifying those that may predict poor graft function and primary non-function. Considering the inferior clinical outcomes reported from DCD liver transplantation, we specifically focussed on identifying whether metabolite features differed between DCD and DBD liver grafts. Biopsies were obtained while the grafts were in an ice bath after a variable period of cold ischaemia had elapsed, and again in the post reperfusion phase. In a limited number of patients biopsied were also obtained in the pre-donation setting. All recipients involved were adequately informed and signed a consent form.

### Clinical data

Overall, a total of 37 (DBD; n = 27 and DCD; n = 10) liver grafts were studied and the clinical courses of the recipients were followed. In DBD donors, the brain stem death criteria had been confirmed prior to the donor operation, which involved dissection and isolation of graft blood vessels while the circulatory function was intact. In DCD donors (n = 10), death was confirmed according to the Institute of Medicine guidelines after obligatory 5 minutes standoff time from the circulatory arrest, after which the donor operation was performed as rapidly as possible [14]. The technical aspects of the organ procurement were otherwise similar. Both in the DBD and DCD procedures, grafts were perfused with Hyper-Osmolar Citrate (HOS, via cannula inserted to the aorta) and University of Wisconsin (UW, via portal vein) preservation fluids. Following donor operations, the liver grafts were packed on ice and transported to the location of the recipient operation, where the grafts were prepared for implantation while immersed in an ice bath. A wide spectrum of procedure related parameters as well as patient demographics and outcome data were recorded for comparison. These included cold ischaemia time (CIT) elapsed prior to the bench biopsy, overall CIT, donor warm ischaemia time (dWIT) in DCD, immediate post operative outcomes, acute physiological status of the recipient while in the intensive care unit, basic liver graft functions, episodes of graft rejection, graft failures and survival outcomes.

Patients underwent OLT for a variety of indications including alcoholic liver disease, primary biliary cirrhosis, primary sclerosing cholangitis, viral hepatitis (HBV/HCV), non-alcoholic steatohepatitis, polycystic disease, Cryptogenic/Autoimmune and Wilson’s disease. The median age of recipient was 53 and 56 years for DCD and DBD respectively. The median (range) model for end-stage liver disease scores of DCD and DBD graft recipients were 12 (8–22) and 16 (6–22) respectively (Table 1).

**Table 1:** Donor and recipient demographics and surgical data.

	DBD (n = 27)	DCD (n = 10)	p-value
Donor gender • Male •Female	• • 11 (41%) •16 (59%)	• • 2 (20%) • 8 (80%)	0.440
Donor age	50.0 (14.2)	49.7 (12.8)	0.948
Donor BMI	26.0 (5.4)	25.4 (3.0)	0.738
Donor cause of death • ICH • Head Injury • Cardiac Arrest • Other	• 18 (67%) • 3 (11%) • 1 (3%) • 5 (19%)	• 4 (40%) • 1 (10%) • 2 (20%) • 3 (30%)	0.294
Graft CIT	484.5 (143.6)	461.0 (117)	0.646
dWIT	-	19.9 (5.7)	
Graft microsteatosis	• None (4) • Mild (22) • Moderate (1)	• None (2) • Mild (8)	
Graft macrosteatosis	• None (2) • Mild (19) • Moderate (3) • Severe (3)	• None (1) • Mild (6) • Severe (3)	
Recipient gender • Male • Female	• 17 (63%) • 10 (37%)	• 5 (50%) • 5 (50%)	0.708
Recipient age	53.7 (9.1)	52.9 (7.3)	0.812
Recipient aetiology • ALD • HCV • PSC • PBC • PCLD • Other	• 9 (33%) • 4 (15%) • 2 (7%) • 4 (15%) • 2 (7%) • 6 (23%)	• 5 (50%) • 2 (20%) • 1 (10%) • 1 (10%) • 1 (10%) • 0	0.681
Presence of HCC	3 (11%)	3 (30%)	0.166
Recipient MELD	16.2 (5.1)	12.3 (4.1)	0.036
Graft Implantation time (minutes)	46.9 (15.9)	40.7 (15.8)	0.296
Operating time (minutes)	276.7 (71.7)	320.2 (98.3)	0.148
Peak AST	1783.5 (2088.2)	3374.7 (2641.3)	0.064
Days on ITU	2.5 (3.0)	5.4 (9.5)	0.160
Length of hospital stay (days)	13.9 (8.5)	14.5 (5.5)	0.825

Values expressed as mean (standard deviation) or number (percentage) as appropriate. Bold values indicate a significant difference (p<0.05) between donor types by ANOVA, t-test or Chi-square as appropriate. ALD = Alcoholic liver disease; AST = aspartate aminotransferase; BMI = Body mass index; CIT = Cold ischaemic time; DBD = Donation after brain death; DCD = Donation after circulatory death; HBV = Hepatitis B; HCC = Hepatocellular carcinoma; HCV = Hepatitis C; ICH = Intracranial haemorrhage; MELD = Model for end-stage liver disease; PBC = Primary biliary cirrhosis; PCLD = Polycystic liver disease; PSC = Primary sclerosis cholangitis; dWIT = Donor warm ischaemic time (DCD only)

Liver samples were obtained by Menghini biopsy needle [11520–19, 19swg (1.0mm) x 70mm; Dixons Surgical Instruments Ltd, Wickford, Essex, UK] for all 37 liver grafts at two stages of OLT: T_1_ –after organ retrieval and transportation to the implanting centre while the liver was prepared on the bench while immersed in an ice bath at a temperature of 0–4°C T1and T_2_ (post reperfusion biopsy) once the graft had been reconnected and the patient haemodynamics stabilised, and usually towards the end of the recipient procedure, after warm ischaemic period and reperfusion injury. In addition, for six DBD grafts an additional biopsy was taken at T_0_, during the donor surgical phase while the liver was still in the donor’s body cavity. There were 80 liver allograft biopsies and these were snap frozen in liquid nitrogen and stored at -80°C until sample preparation for the direct infusion FT-ICR mass spectrometry based metabolomics.

### Direct infusion FT-ICR mass spectrometry based metabolomics

Samples were prepared for the metabolomics analysis as described previously [19]. Briefly, biopsies were extracted using a methanol:chloroform:water method, separating the extracts into polar and non-polar fractions [20]. In total, 80 samples liver biopsy samples were extracted and from these one quality control (QC) sample was prepared by pooling a fraction of each of the 80 extracts (which was then aliquoted into 11 identical fractions). The polar metabolite fraction of each sample was analysed by USA; LTQ FT Ultra) from *m/z* 70 to 590, in positive ion mode, using the SIM-stitching approach [21]. Each sample was analysed in triplicate. To minimise false positive metabolites in the data matrix (due to noise), only peaks present in at least 2 of the 3 replicate measurements of each sample were retained, and then only peaks present in at least 75% of all the samples were retained for further analysis [22]. This data processing also served to exclude any peaks in the mass spectra that arose from the drugs that were known to be administrated to the donors and recipients. The final data matrix consisted of 1260 reproducibly detected peaks (rows) and 91 variables (80 biopsies and 11 quality control samples; columns). The matrix contained 9.29% of missing data which was imputed using a weighted k-nearest neighbours algorithm (*k* = 5) [23]. Data were then normalized using the probabilistic quotient method [24] and subjected to a generalised log transformation (prior multivariate analysis) to stabilise the technical variance across the peaks and hence to avoid the highest abundance peaks from dominating the multivariate analysis [25]. Putative metabolite names were assigned to the peaks based on their mass-to-charge ratio and taking into account commonly detected ions forms, including [M-e]^+^, [M+H]^+^, [M+Na]^+,^ [M+^39^K]^+^, [M+^41^K]+, [M+2Na-H]^+^, [M+2^39^K-H]^+^ and [M+NH_4_]^+^.

### Statistical analyses

Potential clinical differences between DBD and DCD grafts were evaluated by testing each OLT variable, including cold ischaemia time, warm ischaemia time, hours in ITU, number of days-in hospital following OLT, peak aspartate transaminase (AST), and incidence of primary non-function. Non-parametric 2-sample Wilcoxon rank-sum test was applied for continuous numerical variables (e.g., CIT) and Fisher’s exact tests was used for binary variables (e.g., the occurrence of primary non-function). The obtained *p* values were adjusted for multiple hypothesis testing using the Benjamini and Hochberg method to control the false discovery rate [26].

Principal component analysis (PCA) was used to represent the multivariate FT-ICR mass spectral metabolomics data in 2-dimensional space in terms of principal components PC1 and PC2. Univariate statistical analysis, on a per peak basis, was used to discover if any metabolites differed significantly a) between donor (T_0_), cold (T_1_) and post-reperfusion (T_2_) phases across all patients, and b) between DCD and DBD grafts in the cold phase (T_1_) and, separately, post-reperfusion (T_2_) phase. Here, the Anderson-Darling test was used to evaluate normality assumptions, and since ca. 40% of the peaks did not follow a normal distribution, non-parametric statistical methods were used, specifically a two-sample Wilcoxon rank-sum test (DCD and DBD comparison) and its extension to more groups, Kruskal-Wallis one-way analysis of variance (T_0_, T_1_ and T_2_ comparison), both with a Benjamini and Hochberg false discovery correction. In addition, Gain Scores Analysis (Kruskal-Wallis on gain scores) was used to discover those metabolites that changed in a significantly different manner from cold-phase to post-reperfusion between the DCD and DBD grafts. FDR of 5% was used as cut-off values to identify statistically significant features. All statistical analyses were carried out using R version 3.0.2, a free programming language and software for statistical computing and graphics. Putatively identified metabolites were assigned to KEGG metabolic pathways, as defined in the KEGG database [27,28]

## Results and Discussion

### Clinical outcomes

The liver transplantation procedures were carried out in a similar manner for DBD and DCD grafts and we did not notice any significant differences neither at the procedure level nor with the short-term outcomes. The mean CIT was 484.52 ± 143.59 minutes (DBD) and 461.00 ± 116.97 minutes (DCD), whereas the mean implantation time, when grafts were exposed to further warm ischaemia until the circulation was restored, was 41.85 ± 6.94 minutes (DBD) and 41.80 ± 9.77minutes (DCD). The recipients spent on average 95.04 ± 120.80 (DBD) and 123.22 ± 174.48 (DCD) hours in the intensive care unit. The majority of OLTs were successful; three patients in the entire study group had perioperative mortality (n = 2 in the DBD group). The causes of death were related to PNF in two patients (one patient each in the DCD and DBD groups) and related to hepatic artery thrombosis in the third patient (DBD) (Table 1).

### Changes in hepatic metabolism during transplantation

**Fig 1:**
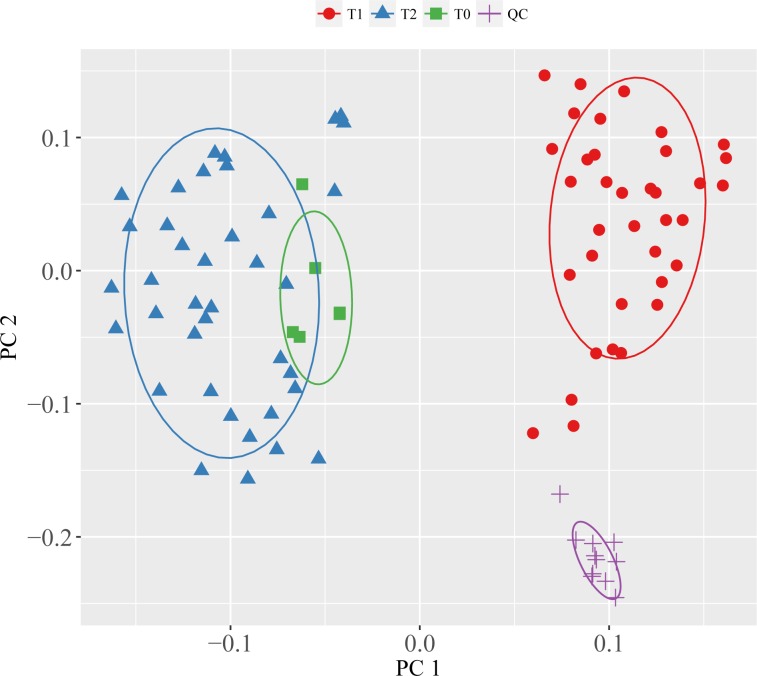
Principal component analysis scores plot showing the similarities and differences between the metabolic profiles of the grafts in donor (T_0_), cold (T_1_) and post-reperfusion phases (T_2_). The variance explained by PC1 = 39.82% and PC2 = 29.58. The close grouping of the donor and post-reperfusion biopsies along with their clear separation from the cold-phase biopsies (along PC1) is further supportive of the rapid resumption of the biochemical functions in the reperfused grafts and shows the direction of metabolic changes through the patient journey and the OLT procedure—from ‘healthy’ donor grafts through cold-phase to almost fully functional grafts post reperfusion.

FT-ICR mass spectra of the extracted biopsies contained 1260 reproducibly detected peaks of which 448 (35.56%) were putatively annotated based upon accurate mass measurements and the Kyoto Encyclopaedia of Genes and Genomes database (S1 Table). Principal component analysis verified the high technical reproducibility of the mass spectra, evidenced by the clustering of the measurements of the QC sample on the PCA scores plot (Fig 1). Furthermore, the PCA scores showed a clear separation between the biopsies from the cold phase (T_1_) and post reperfusion (T_2_). The clustering of the donor biopsies (T_0_; obtained from six DBD grafts while the organs were still perfused with warm circulation) close to the post-reperfusion biopsies (T_2_), with both groups having very distinctive metabolic profiles compared to the biopsies originating from the cold phase sampling is a striking result (Fig 1). This signifies the distinctively different metabolism of hepatocytes in the perfused state compared to those in cold storage. This metabolic separation was confirmed by univariate testing that detected 688 (54.60%) significantly different peaks between T1 and T_2_, 293 peaks (23.25%) between T_0_ and T1, and only 124 peaks (9.84%) between T_0_ and T_2_ (Kruskal-Wallis test, p < 0.05) (Table 2). In our previous proof-of-principle OLT study, we identified a plethora of metabolic responses in the post-reperfused grafts compared to their cold-phase state and concluded that these changes reflected the rapid resumption of the biochemical functions of hepatocytes following reperfusion, including increased urea production, bile acid synthesis and clearance of the preservation solution. Here, in addition to verifying these expected metabolic responses, we observed additional key metabolic changes including, amongst others, putatively annotated essential (threonine and valine) and non-essential (tyrosine, serine and proline) amino acids, taurine (a major constituent of bile), and kynurenine (a central compound in the tryptophan metabolism pathway.

**Table 2:** Top putatively annotated metabolic fold-changes (FC) in the liver grafts between donor phase (T_0_), cold phase (T_1_) and post-reperfusion phase (T_2_), considering the DBD and DCD biopsies as one group. The average absolute ppm error was 0.3631, range: 0.0029–0.9828.

*Putative metabolite*	*m/z (observed)*	*Empirical formula*	*Ion*	FC: T_2_/T_1_	FC: T_2_/T_0_	FC: T_1_/T_0_	*Significance*	*Univariate rank*	*PC1 rank*
Histidine^*^	178.05876	C_6_H_9_N_3_O_2_	Na, H, ^39^K	3.88	1.43	0.37	T_1_ vs. T_2_; T_1_ vs. T_2_	1, 117, 181	1, 130, 198
Malate	157.01079	C_4_H_6_O_5_	Na, 2K-H	4.61	1.64	0.36	T_1_ vs. T_0_; T_1_ vs. T_2_	3, 259	58, 116
Glutamate^*^	170.04244	C_5_H_9_NO_4_	Na, 2Na-H	5.14	1.69	0.33	T_1_ vs. T_0_; T_1_ vs. T_2_	13, 37	15, 20
Serine	128.03181	C_3_H_7_NO_3_	Na, 2Na-H, K(39), H	2.95	1.77	0.60	T_1_ vs. T_0_; T_1_ vs. T_2_	15, 19, 143, 203	101, 105, 111, 205
Glutamine	169.05842	C_5_H_10_N_2_O_3_	Na, ^39^K, 2K-H	4.51	1.41	0.31	T_1_ vs. T_0_; T_1_ vs. T_2_	16, 161, 233	12, 128, 172
N-Acetyl-L-glutamate	212.05305	C_7_H_11_NO_5_	Na	3.60	2.81	0.78	T_1_ vs. T_2_; T_0_ vs. T_2_	18	30
O-Phospho-L-serine	207.99835	C_3_H_8_NO_6_P	Na, H, ^39^K	6.14	1.37	0.22	T_1_ vs. T_0_; T_1_ vs. T_2_	20, 84, 146	23, 56, 152
Tyrosine	204.06321	C_9_H_11_NO_3_	Na, H	3.46	1.32	0.38	T_1_ vs. T_0_; T_1_ vs. T_2_	23, 175	25, 90
AT0P^*^	472.00083	C_10_H_15_N_5_O_10_P_2_	2Na-H, Na	5.46	1.11	0.20	T_1_ vs. T_0_; T_1_ vs. T_2_	26, 51	45, 84
CT0P-choline	489.11491	C_14_H_26_N_4_O_11_P_2_	H	0.42	1.16	2.73	T_1_ vs. T_0_; T_1_ vs. T_2_	49	41
Mannitol^*^	223.04042	C_6_H_14_O_6_	^41^K	0.03	1.34	46.97	T_1_ vs. T_0_; T_1_ vs. T_2_	61	4
Taurine	148.00390	C_2_H_7_NO_3_S	Na	2.11	1.15	0.55	T_1_ vs. T_0_; T_1_ vs. T_2_	88	109
Citrate	193.03435	C_6_H_8_O_7_	H	0.20	2.44	12.14	T_1_ vs. T_0_; T_1_ vs. T_2_	97	92
Threonine	142.04748	C_4_H_9_NO_3_	Na	2.76	1.37	0.50	T_1_ vs. T_2_	110	66
T2oline	138.05256	C_5_H_9_NO_2_	Na, H	2.40	2.75	1.14	T_1_ vs. T_2_; T_0_ vs. T_2_	115, 221	235, 331
GMP	386.04730	C_10_H_14_N_5_O_8_P	Na	3.03	0.98	0.32	T_1_ vs. T_0_; T_1_ vs. T_2_	140	147
Glucose	221.02478	C_6_H_12_O_6_	^41^K	0.38	0.85	2.25	T_1_ vs. T_0_; T_1_ vs. T_2_	153	114
Glycocholate^*^	488.29844	C_26_H_43_NO_6_	Na	4.49	3.27	0.73	T_1_ vs. T_2_	154	170
Succinate	141.01584	C_4_H_6_O_4_	Na	0.49	1.06	2.17	T_1_ vs. T_0_; T_1_ vs. T_2_	185	106
Valine	140.06820	C_5_H_11_NO_2_	Na, ^39^K, H	2.63	1.44	0.55	T_1_ vs. T_2_	186, 218, 253	260, 272, 281
Choline	145.06889	C_5_H_14_NO	^41^K	2.01	2.73	1.36	T_1_ vs. T_2_; T_0_ vs. T_2_	199	377
Formate	90.97661	CH_2_O_2_	2Na-H	5.37	3.47	0.65	T_1_ vs. T_2_	205	582
O-Phospho-L-homoserine	200.03205	C_4_H_10_NO_6_P	H	2.87	1.35	0.47	T_1_ vs. T_2_	209	83
Kynurenine	209.09221	C_10_H_12_N_2_O_3_	H	1.78	2.76	1.55	T_1_ vs. T_2_; T_0_ vs. T_2_	232	338
Aspartate	134.04479	C_4_H_7_NO_4_	H	2.56	1.17	0.46	T_1_ vs. T_0_; T_1_ vs. T_2_	243	139
Urea^*^	98.99550	CH_4_N_2_O	^39^K	2.48	1.31	0.53	T_1_ vs. T_2_	268	308

^*^, metabolic changes observed, verifying those reported in our proof-of-principle study[19]

### Hepatic metabolism in DBD compared to DCD grafts

**Fig 2:**
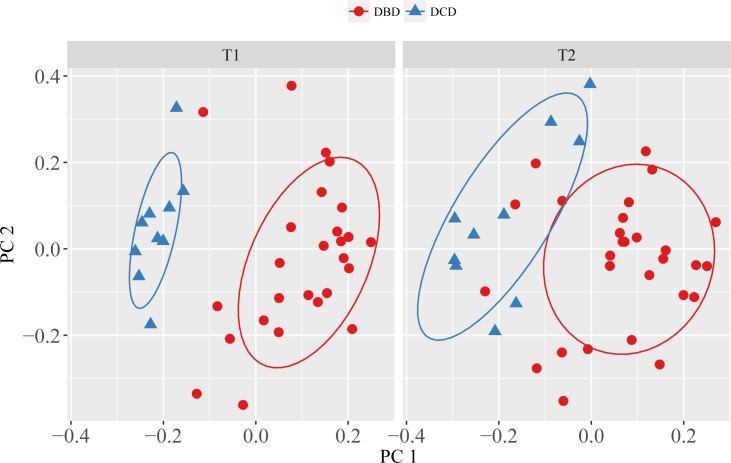
Principal component analysis scores plots highlighting the metabolic separation of the DBD and DCD grafts in the cold phase (T_1_) and separately the post-reperfusion phase (T_2_), based on analyses of just the 50 and 64 peaks identified as being significantly different (between DBD and DCD) for the T_1_ and T_2_ groups, respectively. Variance explained for T_1_, PC1 = 36.75% and PC2 = 19.39% and for T_2_, PC1 = 25.22% and PC2 = 17.41%.

We identified a small subset of peaks that distinguished the DCD and DBD grafts at the metabolic level. In particular, we detected 50 peaks including 11 putatively annotated compounds that differed between DCD and DBD in the cold phase (T_1_), 64 peaks (10 putatively annotated) that differed between DCD and DBD following reperfusion (T_2_), and 72 peaks (10 putatively annotated) that changed from T_1_ to T_2_ in a significantly different manner between DCD and DBD grafts (Table 3). The PCA scores plots, based only on these sub-selections of peaks, provide a visualisation of the clear separation of the DCD vs. DBD grafts along PC1 for the cold-phase biopsies and almost as clear separation for the post-reperfusion biopsies (Fig 2)

**Table 3:** Top putatively annotated metabolic fold-changes between the DCD and DBD grafts: combined results for the comparison, (i) in the cold phase (T_1_), (ii) following reperfusion (T_2_), and (iii) in response from going from T_1_ to T_2_. The average absolute ppm error was 0.2758, range: 0.0069–0.6835.

*Putative metabolite*	*m/z (observed)*	*Empirical formula*	*Ion*	*Fold-change DCD/DBD*^*1*^	*Univariate Rank*^*2*^
Tryptophan	205.09725	C_11_H_12_N_2_O_2_	H	1.88^T^_1_, 1.10^T2^	3^T^_1_, 28^G^
Adenylosuccinate	464.08155	C_14_H_18_N_5_O_11_P	H	0.40^T^_1_	6^T^_1_
GMP	402.02124	C_10_H_14_N_5_O_8_P	^39^K	0.47^T^_1_	15^T^_1_
Malate	210.94066	C_4_H_6_O_5_	2K-H	0.44^T^_1_	18^T^_1_
ADP	465.99284	C_10_H_15_N_5_O_10_P_2_	^39^K	0.41^T^_1_	20^T^_1_
D-Glucose	203.05261	C_6_H_12_O_6_	Na	1.72^T^_1_, 1.22^T2^	22^T^_1_, 9^G^
ADP	450.01895	C_10_H_15_N_5_O_10_P_2_	Na	0.53^T^_1_	25^T^_1_
O-Acetyl-L-carnitine	204.12305	C_9_H_18_NO_4_	-e	0.45^T^_1_	28^T^_1_
Kynurenine	209.09221	C_10_H_12_N_2_O_3_	H	1.80^T^_1_	43^T^_1_
Leucine	132.10190	C_6_H_13_NO_2_	H	1.49^T^_1_	47^T^_1_
Pantothenate	220.11806	C_9_H_17_NO_5_	H	0.60^T^_1_	49^T^_1_
Glutathione	352.05497	C_10_H_17_N_3_O_6_S	2Na-H	1.13^T^_1_, 1.88^T2^	3^T^_2_^,^ 38^G^
Threonine	142.04748	C_4_H_9_NO_3_	Na	1.37^T^_1_, 2.07^T2^	4^T^_2_, 70^G^
Leucine	154.08388	C_6_H_13_NO_2_	Na	1.37^T^_2_	16^T^_2_
Glutamate	170.04244	C_5_H_9_NO_4_	Na	0.99^T^_1_, 1.61^T2^	25^T^_2_, 72^G^
Creatine	154.05872	C_4_H_9_N_3_O_2_	Na	1.45^T^_2_	32^T^_2_
Glutamate	192.02440	C_5_H_9_NO_4_	2Na-H	1.01^T^_1_, 2.78^T2^	38^T^_2_, 45^G^
Threonine	120.06551	C_4_H_9_NO_3_	H	1.37^T^_2_	41^T^_2_
T2oline	138.05256	C_5_H_9_NO_2_	Na	1.59^T^_2_	53^T^_2_
Pantothenate	220.11806	C_9_H_17_NO_5_	H	0.79^T^_2_	62^T^_2_
Leucine	132.10190	C_6_H_13_NO_2_	H	1.32^T^_2_	64^T^_2_
Ornithine	133.09716	C_5_H_12_N_2_O_2_	H	1.98^T^_1_, 1.05^T2^	42^G^
Serine	150.01378	C_3_H_7_NO_3_	2Na-H	0.78^T^_1_, 3.01^T2^	54^G^
SAM	399.14460	C_15_H_22_N_6_O_5_S	H	1.23^T^_1_, 0.68^T2^	51^G^
Glucose	221.02478	C_6_H_12_O_6_	^41^K	1.40^T^_1_, 1.05^T2^	59^G^

^1^ Fold-change calculated for the corresponding phase, cold phase (T_1_) or post reperfusion (T_2_)

^2^ Ranking carried out separately for the three comparisons: in the cold phase (T_1_), post-reperfusion (T_2_) or based on the Gain Scores Analysis (G): capturing the metabolic responses between DCD and DBD grafts from the T_1_ to T_2_

**Fig 3:**
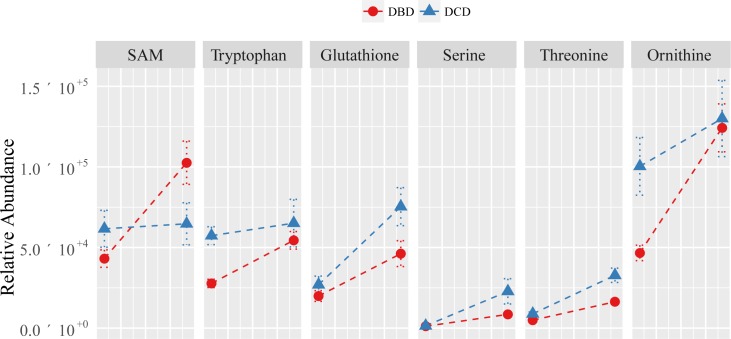
Selected top putatively identified metabolites changed in a significantly different way from the cold phase (T_1_) to post-reperfusion (T_2_) between DCD and DBD grafts. Gain Scores Analysis (Kruskal-Wallis on gain scores) was used to discover those metabolites that changed in a significantly different manner from cold-phase to post-reperfusion between the DCD and DBD grafts.

The key metabolic differences between DBD and DCD grafts in the cold phase (T_1_) included increased levels (in DCD) of the putatively annotated metabolites tryptophan, kynurenine, glucose and leucine and decreased levels of adenylosuccinate, GMP, ADP, malate, O-acetyl carnitine and pantothenate (Table 3). The observed putative metabolites are involved mainly in tryptophan metabolism, purine metabolism, oxidative phosphorylation and a set of carbohydrate metabolic pathways, including the TCA cycle, pyruvate metabolism, glycolysis/gluconeogenesis and the pentose phosphate pathway (Table 4). Among these findings, tryptophan and its metabolism have received earlier attention in the liver transplantation field. Tryptophan is an essential amino acid that, amongst other roles, serves as a precursor of the neurotransmitter serotonin and vitamin B_3_. Histidine-tryptophan-ketoglutarate (HTK) solution, which contains tryptophan to prevent membrane injury, was proposed as an alternative liver preservation solution to the current gold standard, University of Wisconsin solution. Interestingly the organ preservation solutions used in this study cohort (UW solution and Hyper-osmolar citrate/Marshall’s solution) do not have added tryptophan. The systematic review to compare the efficacy and safety of these two solutions did not show overall significant differences, yet in some cases HTK was believed to perform better, especially in terms of biliary tract flush and prevention of biliary complications [29]. Furthermore, tryptophan can be catabolised either via the kynurenine or serotonin pathways, and hence kynurenine was studied previously to investigate tryptophan metabolism in potential cirrhotic liver transplant recipients. The pre-transplant serum levels of kynurenine as well as the kynurenine/tryptophan ratios were positively correlated with the disease severity, while serum levels of tryptophan and serotonin showed no correlation[30]. The significantly higher levels of tryptophan and kynurenine in DCD grafts (vs. DBD grafts) during the cold phase in our study appears to support the previous studies that identified tryptophan metabolism via kynurenine pathways as a key metabolic change in liver transplantation. Although it was not a key objective to analyse biomarkers related to primary non-function in the present study given the small sample size, the two failed allografts due to primary non-function both had abundantly higher levels of tryptophan and kynurenine (Fig 3).

**Table 4:** Metabolic pathways discovered to differ significantly between DCD and DBD grafts, including the associated putatively annotated metabolites in those pathways.

*Metabolic pathway*	*Putative metabolite*	*Description*^*1*^
Tryptophan metabolism	Tryptophan, Kynurenine	Amino acid metabolism; T_1_^H^
Purine metabolism	Adenylosuccinate, GMP, ADP	Nucleotide metabolism; T_1_^L^
Oxidative phosphorylation	ADP	Energy metabolism; T_1_^L^
TCA cycle	Malate	Carbohydrate metabolism; T_1_^L^
Pyruvate metabolism	Malate	Carbohydrate metabolism; T_1_^L^
Glycolysis / Gluconeogenesis	Glucose	Carbohydrate metabolism; T_1_^H^
Pentose phosphate pathway	Glucose	Carbohydrate metabolism; T_1_^H^
Alanine, aspartate and glutamate metabolism	Adenylosuccinate, Glutamate	Amino acid metabolism; T_1_^L^, PR^H^
Glycine, serine and threonine metabolism	Tryptophan, Threonine, Creatine, Serine	Amino acid metabolism; T_1_^H^, PR^H^
Cysteine and methionine metabolism	Glutathione, Serine, SAM	Amino acid metabolism; T_2_^H^ with exception of lower levels of SAM
Arginine and proline metabolism	Glutamate, Creatine, Proline, Ornithine, SAM	Amino acid metabolism; T_2_^H^ with exception of lower levels of SAM
Valine, leucine and isoleucine degradation & biosynthesis	Leucine, Threonine	Amino acid metabolism; T_1_^H,^ PR^H^
Glutathione metabolism	Glutathione, Glutamate, Ornithine	Metabolism of other amino acids; PR^H^
Taurine and hypotaurine metabolism	Glutamate	Metabolism of other amino acids; PR^H^
D-Glutamine and D-glutamate metabolism	Glutamate	Metabolism of other amino acids; PR^H^
Aminoacyl-tRNA biosynthesis	Tryptophan, Leucine, Threonine, Glutamate, Proline, Serine	Translation; T_1_^H^, PR^H^
ABC transporters	Glucose, Leucine, Glutathione, Glutamate, Proline, Ornithine, Serine	Membrane transport; T_1_^H^, PR^H^
Pantothenate and CoA biosynthesis	Pantothenate	Metabolism of cofactors and vitamins; T_1_^L^, PR^L^
Vitamin digestion and absorption	Pantothenate	Digestive system; T_1_^L^, PR^L^
Bile secretion	Glucose, Glutathione	Digestive system; T_1_^H^, PR^H^

^1^, H, higher levels and L, lower levels of putative metabolites in DCD in the corresponding OLT stage (T_1_ or T_2_)

**Fig 4:**
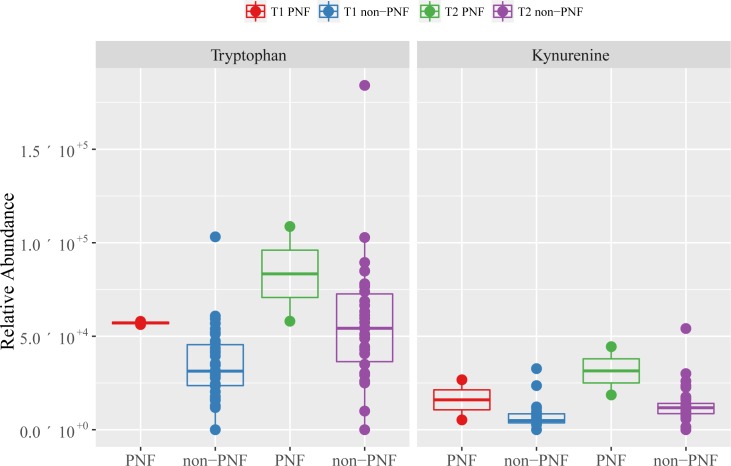
The differences between tryptophan and kynurenine in failed allografts due to Primary non-function/PNF (n = 2) vs. non-PNF (n = 36) in the cold phase and post reperfusion. The data show the relative abundances of the metabolites with 95% confidence intervals (statistics not applied due to limited sample size).

The putatively annotated metabolites that significantly differed between DCD and DBD grafts following reperfusion (T_2_) included increased levels (in DCD) of glutathione, threonine, leucine, glutamate, creatine, glutamate, proline and decreased levels of pantothenate (Table 2). In addition, four of these metabolites including glutathione, threonine, glutamate and glutamate were changed in a significantly different manner in DCD and DBD grafts while they were removed from cold storage (T_1_) and following reperfusion (T_2_). The remaining six metabolites identified as different in the Gain Scores Analyses included tryptophan and glucose (previously observed as significantly different in the cold-phase) as well as ornithine, serine, S-adenosyl methionine (SAM) and glucose (Table 2 and Fig 4). All of these putatively annotated metabolites are primarily involved in amino acid metabolism and translation (aminoacyl-tRNA biosynthesis) and to a lesser extent in metabolism of cofactors and vitamins and bile secretion (Table 3). Interestingly, in DBD grafts, there is a large increase in SAM between the cold phase and post-reperfusion, which does not occur in DCD grafts. While the biochemical implications of this lack of recovery are unknown, we hypothesise that this could affect methylation reactions including DNA methylation, given the importance of this metabolite as a methyl donor [31]. Further targeted investigations of the SAM (and related S-adenosylhomocysteine) metabolic pathway are therefore warranted.

The increased level of glutathione in DCD grafts, which occurs in the cold phase (1.13 times higher in DCD compared to DBD grafts) but is considerably more pronounced (1.88 times higher) following reperfusion, is also an intriguing finding. Glutathione, owing to its thiol group, is known as one of the most effective antioxidants preventing cellular damage caused by reactive oxygen species, as occurs in ischaemia/reperfusion injury. One of its precursors, acetylcysteine, has been studied as a protective molecule in the perioperative treatment of patients undergoing liver transplantation [32]. Our findings show that not only were glutathione levels increased but so were other intermediates of glutathione metabolism such as glutamate and ornithine, indicative of disruption to the glutathione pathway. While glutathione and glutamate shared similar responses at the post-reperfusion time point, i.e. a rapid increase of levels in DCD compared to DBD, ornithine levels were higher in the cold-phase and similar following reperfusion (Fig 4). This could be due to ornithine being used up for the biosynthesis of glutathione via the intermediate by-product of glutamate. These changes may explain the increased oxidative stress incurred by the DCD grafts, owing to the increased ischaemic injury through warm ischaemic damage.

Although metabolomics studies have caused initial enthusiasm in the transplant community as a potential investigative tool to differentiate poorly functioning grafts from those that perform well after transplantation certain limitations have halted the wider applicability. Only one recent publication proposes a metabolic biosignature, based on combination of metabolites altered in liver allografts that perform unsatisfactorily following liver transplantation[4] and this by far the most explicit application of metabolomics in the field of solid organ transplantation. These metabolites responsible for early allograft dysfunction and graft failure include phospholipid metabolism, bile production, ammonia and urea cycles as well as and glutathione metabolism. Interesting some of the metabolites have also been identified in our study, the key metabolite changes related to tryptophan and kynurenine metabolism have not been identified that could be explained by differences in allograft allograft types and analytical techniques involved.

## Conclusions

Previous studies on larger cohorts (n > 300) of DBD and DCD liver transplantations provide evidence for the inferiority of the DCD donations, including increased incidence of primary non-function, biliary complications as well as lower graft- and patient survival [12,33]. Our study has for the first time identified, in un-targeted manner, key metabolic differences between DCD and DBD liver grafts. Since we did not observe any significant differences between the DCD and DBD procedures and outcomes in our study, we believe that these metabolic differences are reflective of the inherent molecular dissimilarities between DCD and DBD grafts. Some of the identified metabolic alterations correlate with our current understanding of the physiological changes surrounding DCD organ donation, including an impact on glucose metabolism by donor warm ischaemia in DCD grafts. However, our observed changes to the tryptophan/kynurenine axis in the DCD grafts are novel findings. Both of these metabolites were observed at ca. 2-fold higher concentration in the DCD grafts (compared to DBD grafts) in the cold phase, suggesting the possibility that these metabolites are responsible for, or at least could be indicators of, the reported higher incidences of increased graft failures in DCD grafts in the literature. In fact, we observed increased levels of tryptophan/kynurenine in allografts with PNF in our study; however, due to few cases we were not able to verify statistically verify this association. Given the role of metabolomics as a hypothesis generating tool, and not to determine whether this metabolic pathway is indeed the cause of graft failure, we conclude that the subsequent clinical investigations of DCD versus DBD transplantations should employ a targeted analytical approach to robustly quantify the metabolites in the tryptophan/kynurenine pathway in the pursuit of more reliable biomarkers of graft function.

## Supporting Information

S1 Table Minimal dataset—FTICR mass spectromtry analysis.xlsx. (XLSX)
